# Risk of sarcopenia, frailty and malnutrition as predictors of postoperative delirium in surgery

**DOI:** 10.1186/s12877-024-05566-1

**Published:** 2024-11-27

**Authors:** Henriette Louise Moellmann, Eman Alhammadi, Soufian Boulghoudan, Julian Kuhlmann, Anica Mevissen, Philipp Olbrich, Louisa Rahm, Helmut Frohnhofen

**Affiliations:** 1 Cranio-and-Maxillo Facial Surgery, University Hospital Düsseldorf, Moorenstraße 5, 40225 Düsseldorf, Germany; 2 Heinrich-Heine-Universität Düsseldorf, Universitätsstraße 1, 40225 Düsseldorf, Germany; 3 Orthopedics and Trauma Surgery, University Hospital Düsseldorf, Moorenstraße 5, 40225 Düsseldorf, Germany

**Keywords:** POD, Surgery, Malnutrition, Geriatric assessment

## Abstract

**Background:**

The risk factors for postoperative delirium are numerous and complex. One approach to identifying patients at risk is to evaluate their nutritional status. The aim of this prospective study is to better understand nutrition as a potential risk factor for postoperative delirium.

**Methods:**

A comprehensive preoperative assessment (Clinical Frailty Scale (CFS), the SARC-F questionnaire, Mini Nutritional Assessment-Short Form (MNA-SF)) were carried out as a prospective clinical study on 421 patients (70+) from 4 different surgical disciplines. Postoperatively, patients are examined daily for the presence of delirium using the 4AT screening tool (Arousal, Attention, Abbreviated Mental Test − 4, Acute change), the Nursing Delirium Screening Scale (NuDesc) and the Confusion Assessment Method (CAM) with its adaptation for the intensive care unit (CAM-ICU).

**Results:**

If there were indications of frailty or sarcopenia in the CFS or SARC-F, the association with delirium was increased 5.34-fold (OR of 5.34 [95% CI: 2.57;11.1]) and 5.56-fold (OR of 5.56 [95% CI: 2.97;10.4]) respectively. Delirium also occurred significantly more frequently with the risk of malnutrition or manifest malnutrition (MNA-SF) than with a normal nutritional status.

**Conclusions:**

Patients’ preoperative and nutritional status significantly impact the risk of developing postoperative delirium. Factors such as frailty, sarcopenia and possible malnutrition must be considered when implementing an effective and targeted preoperative assessment.

**Trail registration:**

German Clinical Trials Registry at https://www.drks.de/DRKS00028614, Registered 25 March 2022.

**Supplementary Information:**

The online version contains supplementary material available at 10.1186/s12877-024-05566-1.

## Introduction

Postoperative delirium (POD) is an acute and transient organ failure of the brain associated with disturbances of perception, consciousness, and attention. It is typically manifesting within the first postoperative week [1]. The prevalence varies between 11.1% and 45.6%, influenced by patient and surgical factors [2]. The occurrence of POD is often associated with an increased complication rate, a more extended hospital stay, higher healthcare costs and increased institutionalization [3–5]. As the disease progresses, there is a higher incidence of repeated hospitalization, functional impairment, elevated mortality rates and an increased risk of dementia [6, 7]. It is assumed that 30–40% of delirium could be prevented [8, 9].Therefore, it is essential to develop implementable and adequate strategies for prevention and treatment to improve patient outcomes and prognoses. Many risk factors for delirium have already been identified using validated screening tools [10, 11].

One aspect of these screenings is the patient’s nutrition. Malnutrition, frailty and low body mass index have been linked to POD [11–13]. Therefore, the early preoperative identification of patients at risk of malnutrition could be an essential aspect of a preoperative assessment to prevent POD [14, 15]. Malnutrition is defined as an altered body composition and cell mass due to an insufficient supply of nutrients, illnesses, and the aging process. In a state of malnutrition, cognitive and physical performance is impaired, adversely affecting clinical outcomes [16–19]. In older patients, the incidence is assumed to be 0.8–24.6% [16, 20]; in surgical patients, the incidence is estimated at 45% [21]. Another aspect is sarcopenia, a muscle disease (muscle wasting) that often occurs in older adults, but can also occur earlier in life [21]. The presence of sarcopenia increases the risk of falls and fractures [22, 23], impairs the ability to perform activities of daily living [24], and is associated with heart disease [25], respiratory disease [26] and cognitive impairment [27]. In addition, sarcopenia leads to impaired mobility [28], reduced quality of life [29], loss of independence or the need for placement in a long-term care facility [30–32] and death [33]. Frailty, a frequent aging disease marked by immense loss of function and increased susceptibility to internal and external stressors, is another significant contributor. In a variety of clinical settings, frailty raises the risk of falls [34], cognitive impairment [35], depression [36], death [37, 38], delirium [39] and comorbidities [40].

The aim of this prospective study is to assess the risk of malnutrition, sarcopenia and possible frailty as potential risk factors for postoperative delirium. The identified potential risk factors will be examined for their association with an increased risk of delirium in different surgical disciplines.

## Materials and methods

The research protocol for the present study received approval from the Ethics Committee of the Medical Faculty of Heinrich Heine University Düsseldorf (study no.: 2022 − 1810). Prior to recruiting the first patients, the research project was registered in a publicly accessible database according to DvH2013, § 35 (German Register of Clinical Studies, DRKS-ID: DRKS00028614). This cohort included older (70+) patients who presented for elective or emergency surgery at the departments for Oral and Maxillofacial Surgery, Orthopedic and Trauma Surgery, General Surgery and Vascular Surgery. Patients who did not consent to participate, had an unstable clinical situation, patients requiring palliative care or with advanced dementia (Reisberg VI-VII) were excluded. An extensive geriatric assessment was carried out on 421 patients (*n* = 267 elective, *n* = 134 emergency patients, *n* = 20 others) on admission after informed consent was obtained. In addition to age, gender, BMI, height and body weight, skinfold thickness, grip strength, comorbidities were also documented. The preoperative assessment included the Clinical Frailty Scale (CFS), SARC-F questionnaire (Strength, Assistance with walking, Rise from a chair, Climb stairs, and Falls), and Mini Nutritional Assessment-Short Form (MNA-SF). The CFS assesses comorbidity, function and cognition to determine a frailty score ranging from 1 (very fit) to 9 (terminally ill) [41]. The 5 components of the SARC-F are each assigned a value between 0 and 2, resulting in a total value between 0 and 10. A value ≥ 4 shows low sensitivity but high specificity in the detection of adult sarcopenia [42–44]. The short form of the MNA, the MNA-SF, is a sensitive and specific instrument for assessing the nutritional status of older people [45]. Up to 14 points can be achieved, with 0–7 points indicating malnutrition, 8–11 points indicating a risk of malnutrition and 12–14 points indicating a normal nutritional status [46]. During the postoperative course, patients were screened daily for the presence of delirium using the Confusion Assessment Method (CAM) along with its Intensive care unit adaptation (CAM-ICU). The delirium screening was carried out by trained nursing staff, attending physicians or doctoral students. In the intensive care unit, screening was carried out every 8 h in the morning and evening in the normal ward. Screening was carried out in the first seven postoperative days or until discharge.

### Statistical analysis

The values obtained from the measurements and the clinical data were analyzed using Jamovi (version 1.6.9, (Computer Software, retrieved from https://www.jamovi.org, accessed on 19 March 2022, Sydney, Australia)). A p-value of 0.05 was set for the hypothesis test [47, 48]. Mean differences are tested with independent T-Test (t) when significant outliers, identified with boxplots were excluded, normal distribution of the dependent variable, tested with Shapiro-Wilk-Test and homoscedasticity, tested with Levene’s test, were met. Mean Differences of non-normal dependent variable data is analyzed with Mann-Whitney-U test (U). Bivariate relationships between relevant variables and the development of delirium (delirium vs. no delirium) were analyzed with chi-square tests. We estimated the odds ratios (OR) of the associations with 95% confidence intervals (CI). Statistical tests were two-sided, and significance was assessed at the alpha level of 0.05. A *p*-value of < 0.05 was defined as significant, a value of < 0.01 as very significant, and a value of < 0.001 as highly significant. A significance level of *p* > 0.05 was set for hypothesis testing. For multiple testing, an adjustment was made by Bonferroni correction to reduce the risk of a type I error [49]. Binomial logistic regression analysis was used to identify influencing factors on delirium that were considered statistically relevant at a significance level of *p* = 0.05.

## Results

The sample comprises 421 patients who underwent surgical procedures under general anesthesia in the above-mentioned surgical departments during the survey period and agreed to participate in this prospective study. The data of 199 women and 222 men aged 79.7 ± 6.5 years are analyzed. The delirium rate of the entire collective (*n* = 416) is 12.3% (*n* = 51). An overview of the master data and initial parameters can be obtained from the supplementary material are shown in the following table (Supplementary Table 1). A extensive assessment and documentation of the patient’s baseline health were performed in the preoperative phase. The parameters captured, and their correlation with the occurrence of delirium were analyzed (Supplementary Table 2). In the cohort, 267 patients (66.6%) were admitted electively and 134 patients (33.4%) were admitted as emergencies. The distribution in the individual specialist disciplines and the delirium rates are shown in the supplementary material (see Supplementary Table 3). An overview of the most common diagnoses and surgical procedures is shown in the supplementary material (see Supplementary Tables 4 and 5).

Upon evaluating the Clinical Frailty Scale (CFS), it was found that 34.6% of the patients (*n* = 99) displayed characteristics indicative of frailty, whereas 65.4% of the patients (*n* = 187) displayed no frailty characteristics. Within the group of patients showing frailty characteristics, the incidence of delirium was 27.3% (*n* = 27), and in the other group, 6.6% (*n* = 12). In frail patients, the association with delirium was 5.34 times higher with an OR of 5.34 [95% CI: 2.57;11.1] and significant with Χ^2^(1) = 23.1, p_Bonferroni_ = 0.007, Cramer’s V = 0.286 (see Supplementary Fig. 1a). The SARC-F questionnaire was used to check for possible sarcopenia. Sarcopenia was probable in 29.1% (*n* = 118) and not in 70.9% (*n* = 288). The delirium rate was 64% (*n* = 32) and 36% (*n* = 18) respectively, with a significant association with Χ^2^(1) = 33.5, p_Bonferroni_ = 0.007, Cramer’s V = 0.289. With an OR of 5.56 [95% CI: 2.97;10.4], the risk of developing a delirium was 5.56 times higher. (see Supplementary Fig. 1b). The MNA-SF was used to evaluate the nutritional status. In 140 patients (35.2%), the nutritional status was normal; in 189 patients (47.5%), there was a risk of malnutrition; and in 69 patients (17.3%), malnutrition was manifest. 64.8% of patients thus show an abnormality. With normal nutritional status, the delirium rate was 2.9% (*n* = 4), with the risk of malnutrition at 16% (*n* = 30) and with manifest malnutrition at 18.8% (*n* = 13). A significant correlation was shown with Χ^2^(2) = 16.5, p_Bonferroni_ = 0.007, Cramer’s V = 0.205 (see Supplementary Fig. 1c). Additional information regarding the occurrence of delirium in elective and emergency patients can be retrieved from the Supplementary material (Supplementary Tables 6–8).

The results of the skinfold thickness and grip strength measurements are listed in the Supplementary material (Supplementary Table 3). The Mann-Whitney U test was conducted to assess whether there were differences in skinfold thickness or grip strength between the two groups about the occurrence of delirium. The distributions of the two groups were different, as indicated by the Kolmogorov-Smirnov test, *p* < 0.05. A significant difference in right and left grip strength was found between the groups (right: U = 5015, p_Bonferroni_ = 0.007, *r* = 0.319 (), left: U = 5230, p_Bonferroni_ = 0.224, *r* = 0.208). Depiction of the parameters regarding delirium is found in the supplementary Material (see Supplementary Fig. 2).

A binomial logistic regression was performed to determine the effect of CFS, SARC-F, MNA-SF as well as grip strength (right and left) and predict the likelihood of developing delirium. The binomial logistic regression model was statistically significant, χ²(5) = 22.8, *p* < 0.001, resulting in a large amount of explained variance, as shown by Nagelkerke’s R² = 0.187. The overall percentage of accuracy in classification was 88.7%, with a sensitivity of 11.5% and a specificity of 99.5%. Of the five variables entered into the regression model, one contributed significantly in predicting delirium: CFS (*p* = 0.039), while the other variables showed no significant effect: SARC-F (*p* = 0.536), MNA-SF (*p* = 0.710) nor grip strength (right: *p* = 0.780, left: *p* = 0.724). The CFS increased the likelihood of developing delirium, OR = 1.54 (95%-CI[1.022, 2.332]). All model coefficients and odds can be found in Supplementary Table 9.

## Discussion

In the study at hand, patients over 70 years of age from various disciplines were included, and the influence of sarcopenia, frailty and nutritional status on the occurrence of delirium was investigated. The present heterogeneous collective shows an overall delirium rate of 12.3% and in the individual disciplines of 7–29%. These results are also reflected in recent literature, with 8.2% [50] in elective and 21.7% [51] in emergency orthopedic and trauma surgery, with 3–37% [52–55] oral and maxillofacial surgery, with 5–39% [56] vascular surgery. Delirium rates of 5–52% [4, 57, 58] are reported for non-cardiac surgery. With a small sample size of 26 patients, we were unable to detect any delirium.

Frailty is a clinical condition characterized by a decrease in a person’s homeostatic reserves while being responsible for increased susceptibility to endogenous or exogenous stress factors or both. The CFS, as a well-validated screening tool, shows a frailty prevalence of 34.6% within our collective. The literature describes a prevalence of 18.6–56% [59]. Other studies have established a link between frailty and delirium [39, 59–61], aligning with our group’s 5.34-fold increase in delirium risk. The integration of frailty assessment into preoperative care is therefore of great relevance, as the CFS increased the likelihood of developing delirium, OR = 1.54 (95%-CI[1.022, 2.332]).

However, the Geriatric Medicine Research Collaborative showed that delirium is less likely to be recognized by the clinical team when the patient is more frail [62]. This highlights the need for proper screening and diagnosis for the presence of delirium even after the identification of patients at risk [39].

Sarcopenia in older patients is widespread and a well-known risk factor for postoperative complications [63–66]. Zucchelli et al. (2017) revealed that 73.6% of emergency-admitted patients over 65 years exhibit low skeletal muscle mass. They also demonstrated a significant association between low skeletal muscle mass and delirium [67]. Low muscle mass and sarcopenia are closely related to the above-mentioned frailty [68, 69]. Both are considered risk factors for delirium. In particular, the change in body composition and loss of muscle mass might lead to an increased risk of adverse drug reactions [70]. In combination with the polypharmacy often present in mature age, this could potentially lead to a higher risk of delirium [71]. In addition to numerous other aspects, the recording of sarcopenia is of central relevance and can easily be realized as well as implemented using SARC-F. In the assessment of nutritional status, a high proportion of patients at risk of malnutrition (*n* = 189; 47.5%) or with manifest malnutrition (*n* = 69; 17.3%) was particularly noticeable. Similar high rates have been reported in other studies in the “acute hospital setting” [3, 72–75] and among the population over 65 [76]. In our study, we refer to a heterogeneous collective from different surgical disciplines with an overall delirium rate of 16–18% at risk of malnutrition or with manifest malnutrition. As this work is a prospective observational study, we cannot establish a causal relationship between malnutrition and the occurrence of delirium. Many other studies have demonstrated a significant link between malnutrition and delirium [12, 13, 77]. We thus recommend that the assessment of nutritional status should be evaluated in the preoperative geriatric assessment. In patients at risk of malnutrition or manifest malnutrition, perioperative nutritional intervention has been described as part of the multidisciplinary approach to prevent delirium [78–80].

Besides nutritional status, numerous clinical and epidemiological studies have also shown that grip strength is highly predictive of short- and long-term mortality and morbidity [81–84]. We were able to demonstrate significant differences in grip strength between patients with delirium and those without. Reduced grip strength in patients indicates increased post-surgical complications, extended hospital stays, higher rates of rehospitalization, and a decline in physical condition [81]. Matos et al. (2007) highlighted handgrip strength measurement as an effective initial screening tool in hospitals for identifying patients at risk of malnutrition [82].

Even though grip strength showed a significant association with the occurrence of delirium, it can still be assumed that the measurement of skinfold thickness might also be a good indicator of malnutrition [85] and thus increase the risk of delirium. Sahoo et al. (2022) showed a triceps skinfold thickness (TST) with a cut-off value of 13.5 cm, a 98.1% sensitivity, and 93.3% specificity for detecting malnutrition [85].

As with all research, certain limitations apply. Despite the relatively high number of patients included in our study, the sample size is somewhat dissatisfying. Recruitment was difficult in some disciplines, as there were significantly fewer patients over the age of 70 and the rate of test refusals was higher. Approximately one third of patients who met the inclusion criteria refused to participate in the study, which represents a potential source of bias. And, there remains a risk that patients are not willing to be tested due to existing masked cognitive impairment. In addition to the prospective study design, the use of validated instruments is a major strength of our study. In particular, these approaches are clinically relevant, time-saving and practical to use without the need for extensive training.

The relevance and challenges of comprehensive geriatric assessment are becoming increasingly relevant as the number of older people and the complexity of operations performed rises. Personalized medicine is a patient-centered approach for holistic treatment and considers all factors that influence the patient’s condition and clinical outcome [86]. It is reasonable to assume that a preoperative comprehensive geriatric assessment has a positive impact on postoperative outcomes in elderly patients undergoing elective surgery [87]. How a geriatric assessment might be performed within the surgical disciplines can be controversially discussed [88]. As each discipline has varying requirements and challenges for and to patients, the integration of a comprehensive geriatric assessment into the daily routine remains demanding. It is also crucial to recognize that nutrition is influenced differently in individual disciplines due to specific circumstances. In trauma and orthopedic surgery, factors such as immobility, reduced energy consumption, loss of appetite, constipation, and other gastrointestinal symptoms are prevalent. In Maxillofacial surgery, for example, issues include the lack of ability for oral nutrition, reliance on parenteral nutrition or liquid diets, difficulty in ingesting food, swallowing problems, and intraoral swelling, among others. These various factors should be considered as well. There is a need for a specialized assessment with objective and targeted instruments to identify at-risk patients and use resources efficiently.

Chen et al. showed in 2017 that targeted programs such as Hospital Elder Life Program (HELP) [89] or modifications [80] could reduce the odds for delirium by 56% and shorten LOS in abdominal surgery [80, 89]. Such programs, perioperative nutritional support [90] and also prehabilitation [91], should be investigated as relevant pillars of delirium prevention in further studies.

## Conclusion

The preoperative general health and nutritional status of patients significantly influence the risk of developing postoperative delirium. When implementing an effective and targeted preoperative assessment, key factors such as frailty, sarcopenia, and malnutrition must be considered. This comprehensive approach can help identify at-risk patients for delirium, enabling targeted interventions to reduce the incidence of postoperative complications and improve postoperative outcomes.

## Electronic supplementary material

Below is the link to the electronic supplementary material.

Supplementary Material 1

## Data Availability

The datasets used and/or analysed during the current study are available from the corresponding author on reasonable request.

## Abbreviations

ASA: American Society of Anesthesiologists
BMI: Body mass index
CAM: Confusion Assessment Method
CAM-ICU: Confusion Assessment Method- Intensive Care Unit
CI: Conficende interval
CFS: Clinical Frailty Scale
DRKS: German Clinical Trials Registry
HELP: Hospital Elder Life Program
ICU: Intensive Care Unit
MNA-SF: Mini Nutritional Assessment Short-Form
Nu-Desc: Nursing Delirium Screening Scale
OR: Odds Ratio
PAD: Peripheral artery disease
POD: Postoperative delirium
SARC-F: Strength, Assistance with walking, Rise from a chair, Climb stairs and Falls
TEP: Total endoprosthesis
TEA: Thrombendarteriectomy
TST: Triceps skinfold thickness
WHO: World Health Organization
