# Supplementary material for: Risk of sarcopenia, frailty and malnutrition as predictors of postoperative delirium in surgery
Source: BMC Geriatr. 2024 Nov 27;24:971. doi: 10.1186/s12877-024-05566-1 (PMC11600611; doi:10.1186/s12877-024-05566-1)
Supplement: Supplementary file 1 — Supplementary Material 1 [file 12877_2024_5566_MOESM1_ESM.docx]

**Supplementary Material**

Supplementary Table 1: Overview descriptives

| **Gender** | **Male** | **Female** |
| --- | --- | --- |
| n=421 | 52.7% (n= 222) | 47.3% (n=199) |
| **Age n=419** | 78.8 ± 6.2 | 80.8 ± 6.7 |
| **height** | 173.0 ±9.25 cm | 164 ± 13.7 cm |
| **Body weight** | 78.5 ± 14.4 kg | 68.0 ± 15.2 kg |
| **BMI** | 25.7 ± 3.86 | 24.4 ± 5.46 |

Supplementary Table 2: Overview ASA-Classification about the delirium rate

|  |  |  | Delirium |  |  |
| --- | --- | --- | --- | --- | --- |
| **ASA (n=399)** |  |  | No | Yes |  |
| **I** | n=17 | 4.3% | 16 | 1 | 5.9% |
| **II** | n= 131 | 32.8% | 125 | 4 | 3.1% |
| **III** | n= 227 | 56.9% | 189 | 36 | 15.9% |
| **IV** | n= 24 | 6.0% | 16 | 7 | 29.2% |

Supplementary Table 3 Presentation of the distribution of patients in the individual disciplines and the respective delirium rates.

| **Surgical discipline** | **elective/**  **emergency** | **n** | **% of total** | **Delirium** | **n** | **%** |
| --- | --- | --- | --- | --- | --- | --- |
| **Orthopaedic and**  **Trauma Surgery** | **elective** | 46 | 11.5 % | No | 40 | 90.9 % |
|  |  |  |  | Yes | 4 | **9.09 %** |
|  | **emergency** | 111 | 27.7 % | No | 90 | 81.81 % |
|  |  |  |  | Yes | 20 | **18.18 %** |
| **Oral and Maxillofacial Surgery** | **elective** | 83 | 20.7 % | No | 77 | 92,77 % |
|  |  |  |  | Yes | 6 | **7.22 %** |
|  | **emergency** | 7 | 1.7 % | No | 5 | 71.43 % |
|  |  |  |  | Yes | 2 | **28.57 %** |
| **Vascular Surgery** | **elective** | 111 | 27.7 % | No | 102 | 91.89 % |
|  |  |  |  | Yes | 9 | **8.11 %** |
|  | **emergency** | 16 | 4.0 % | No | 12 | 75.00 % |
|  |  |  |  | Yes | 4 | **25.55 %** |
| **General Surgery** | **elective** | 27 | 6.7 % | No | 26 | 0.0 % |
|  |  |  |  | Yes | 0 | **0.0 %** |
|  | **emergency** | 0 | 0.0 % | No | 0 | 0.0 % |
|  |  |  |  | Yes | 0 | **0.0 %** |

Supplementary Table 4: Overview of the main diagnoses within the individual specialist disciplines.

| **Main diagnosis** | **Counts** | **% of total** |
| --- | --- | --- |
| **Orthopaedic and  Trauma Surgery** |  |  |
| Accident | 33 | 7.9 % |
| Orthopaedic issue | 131 | 31.4 % |
| **Oral and Maxillofacial Surgery** |  |  |
| Dentoalveolar | 13 | 3.1 % |
| Osteonecrosis | 15 | 3.6 % |
| Infection | 1 | 0.2 % |
| Trauma | 13 | 3.1 % |
| Tumor | 46 | 11.0 % |
| **Vascular and General Surgery** |  |  |
| Aneurysma | 34 | 8.2 % |
| PAD(peripheral artery disease) | 34 | 8.2 % |
| Stenosis | 28 | 6.7 % |
| Hernia | 12 | 2.9 % |
| Stoma | 3 | 0.7 % |
| Woundhealing disorder | 20 | 4.8 % |
| Malignoma | 16 | 3.8 % |
| others | 18 | 4.3 % |

Supplementary Table 5: Overview of the type of surgeries within the individual specialist disciplines.

| **Type of surgery** | **Counts** | **% of total** |
| --- | --- | --- |
| **Orthopaedic and  Trauma Surgery** |  |  |
| shoulder prothesis | 6 | 1.5 % |
| Hip-TEP (total endoprosthesis) | 43 | 10.5 % |
| Lavage | 4 | 1.0 % |
| Osteosynthesis | 2 | 0.5 % |
| Medullary Nail | 19 | 4.6 % |
| vertebral fixateur | 14 | 3.4 % |
| plate removal | 16 | 3.9 % |
| Wound debridement | 35 | 8.5 % |
| Kyphoplasty | 6 | 1.5 % |
| Knee-TEP | 11 | 2.7 % |
| **Oral and Maxillofacial Surgery** |  |  |
| dentoalveolar surgery | 14 | 3.4 % |
| Decortication | 14 | 3.4 % |
| Wound management | 2 | 0.5 % |
| Biopsy/Panendo | 10 | 2.4 % |
| Osteosynthesis | 36 | 8.8 % |
| Tumor resection | 18 | 4.4 % |
| Reconstruction | 10 | 2.4 % |
| **Vascular Surgery** |  |  |
| vascular Stents | 31 | 7.6 % |
| TEA (thrombendarteriectomy) | 33 | 8.0 % |
| vascular prothesis | 8 | 2.0 % |
| Amputation | 10 | 2.4 % |
| Aneurysma | 1 | 0.2 % |
| Bypass | 3 | 0.7 % |
| **General Surgery** |  |  |
| laparoscopic surgery | 7 | 1.7 % |
| Herniotomy | 9 | 2.2 % |
| Tumorresection | 3 | 0.7 % |
| Thoracoscopic surgery | 5 | 1.2 % |
| Stoma | 2 | 0.5 % |
|  |  |  |
| others | 38 | 9.3 % |

Supplementary Figure 1: Overview of the evaluation of a) CFS vs. Delirium, b) SARC-F vs. Delirium and c) MNA-SF vs. Delirium about the occurrence of delirium.


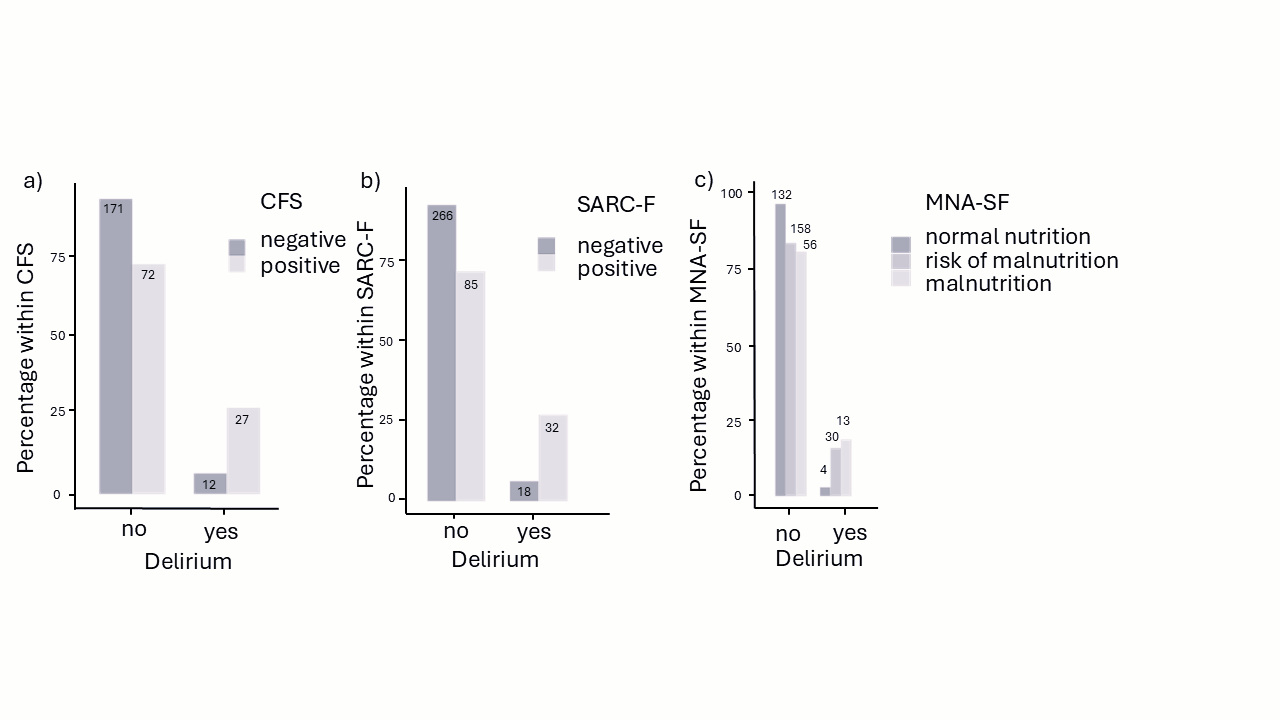


Presentation of delirium rates in patients with a) characteristics indicating frailty in CFS; the association with delirium was 5.34 times higher with an OR of 5.34 [95% CI: 2.57;11.1]; b) sarcopenia, assessed with SARC-F; with an OR of 5.56 [95% CI: 2.97;10.4], the association with delirium was 5.56 times higher; c) impaired nutritional status; With normal nutritional status, the delirium rate was 2.9% (n=4), with the risk of malnutrition at 16% (n=30) and with manifest malnutrition at 18.8% (n=13). A significant correlation was shown with Χ2(2)=16.5, p<0.001, Cramer's V=0.205

Supplementary Table 6: Results of CFS related to the occurrence of delirium in elective and emergency patients.

| **CFS** | **elective/**  **emergency** | **n** | **% of total** | **Delirium** | **n** | **%** |
| --- | --- | --- | --- | --- | --- | --- |
| **no frailty** | **elective** | 124 | 46.2% | No | 118 | 95.16% |
|  |  |  |  | Yes | 6 | **4.84%** |
|  | **emergency** | 53 | 19.7% | No | 47 | 88.68% |
|  |  |  |  | Yes | 6 | **11.32%** |
| **possible frailty** | **elective** | 31 | 11.6% | No | 26 | 83.87% |
|  |  |  |  | Yes | 5 | **16.13%** |
|  | **emergency** | 60 | 22.4% | No | 44 | 73.33% |
|  |  |  |  | Yes | 16 | **26.67%** |

Supplementary Table 7: Results of SARC-F related to the occurrence of delirium in elective and emergency patients.

| **SARC-F** | **elective/**  **emergency** | **n** | **% of total** | **Delirium** | **n** | **%** |
| --- | --- | --- | --- | --- | --- | --- |
| **no sarcopenia** | **elective** | 209 | 54.3% | No | 199 | 95.21% |
|  |  |  |  | Yes | 10 | **4.78%** |
|  | **emergency** | 65 | 16.9% | No | 57 | 87.69% |
|  |  |  |  | Yes | 8 | **12.31%** |
| **possible sarcopenia** | **elective** | 48 | 12.5% | No | 40 | 83.33% |
|  |  |  |  | Yes | 8 | **16.67%** |
|  | **emergency** | 63 | 16.4% | No | 45 | 71.48% |
|  |  |  |  | Yes | 18 | **28.57%** |

Supplementary Table 8: Results of MNA-SF related to the occurrence of delirium in elective and emergency patients.

| **MNA-SF** | **elective/**  **emergency** | **n** | **% of total** | **Delirium** | **n** | **%** |
| --- | --- | --- | --- | --- | --- | --- |
| **normal nutritional status** | **elective** | 102 | 27.0% | No | 100 | 98.04% |
|  |  |  |  | Yes | 2 | **1.96%** |
|  | **emergency** | 30 | 7.9% | No | 28 | 93.33% |
|  |  |  |  | Yes | 2 | **6.67%** |
| **risk of malnutrition** | **elective** | 122 | 32.4% | No | 107 | 87.70% |
|  |  |  |  | Yes | 15 | **12.30%** |
|  | **emergency** | 61 | 16.2% | No | 47 | 77.05% |
|  |  |  |  | Yes | 14 | **22.95%** |
| **malnutrition** | **elective** | 32 | 8.5% | No | 31 | 96.88% |
|  |  |  |  | Yes | 1 | **3.12%** |
|  | **emergency** | 30 | 8.0% | No | 23 | 76.67% |
|  |  |  |  | Yes | 7 | **23.33%** |

*Supplementary Figure 2: Evaluation of triceps skinfold thickness and grip strength measurements.*


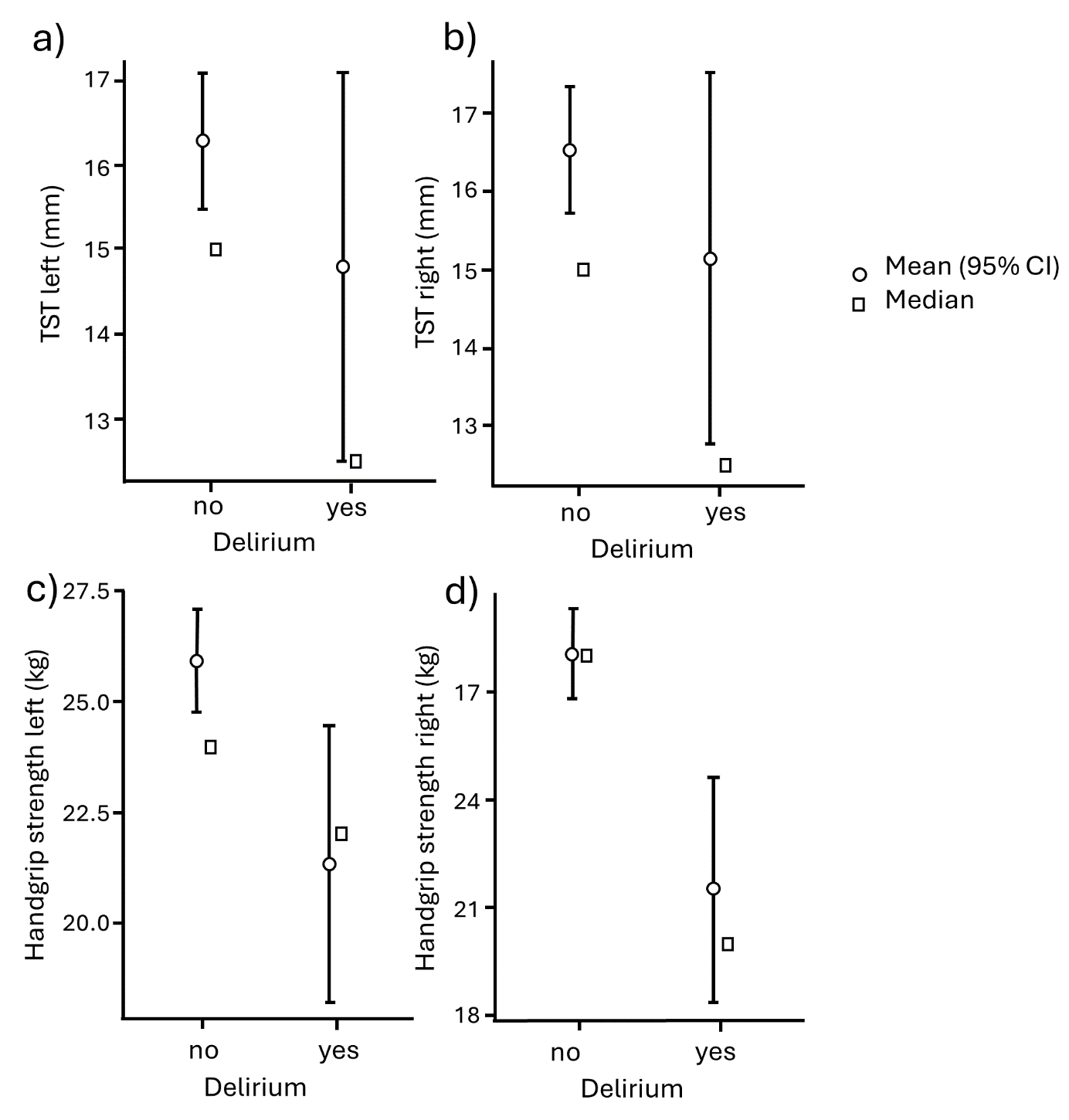


a) Triceps skinfold thickness measurement on the left side. b) Triceps skinfold thickness measurement on the right side. c) Grip strength measurement on the left-hand side, with delirium occurrence. d) Grip strength measurement on the right-hand side, with delirium occurrence.

*Supplementary Table 9. Overview Model Coefficients: Models for predictability of the preoperative parameters (CFS, SARC-F, MNA-SF, Grip strength left and right) of delirium. *Note. The cut-off value is set to 0.5.*

| Model Fit Measures | | | | | | | |
| --- | --- | --- | --- | --- | --- | --- | --- |
|  | | | | | **Overall Model Test** | | |
| **Model** | **Deviance** | **AIC** | **BIC** | **R²N** | **χ²** | **df** | **p** |
| 1 | 141 | 153 | 174 | 0.187 | 22.80 | 5 | < .001 |
| Model Coefficients – Transplant failure yes/no | | | | | | | |
|  | | | | | | **95% Confidence Interval** | |
| **Predictor** | **Estimate** | **SE** | **Z** | **p** | **Odds ratio** | **Lower** | **Upper** |
| Intercept | -30.049 | 14.791 | -2.032 | 0.042 | 0.0495 | 0.00273 | 0.900 |
| CFS | 0.4345 | 0.2104 | 2.065 | 0.039 | 15.442 | 102.242 | 2.332 |
| SARC-F | 0.0645 | 0.1042 | 0.618 | 0.536 | 10.666 | 0.86950 | 1.308 |
| MNA-SF | -0.0375 | 0.1008 | -0.372 | 0.710 | 0.9632 | 0.79059 | 1.173 |
| left (kg) | -0.0182 | 0.0516 | -0.353 | 0.724 | 0.9819 | 0.88752 | 1.086 |
| right (kg) | -0.0147 | 0.0527 | -0.280 | 0.780 | 0.9854 | 0.88871 | 1.093 |
| **Note. Estimates represent the log odds of "Delirium = yes" vs. "Delirium = no"* | | | | | | | |
